# Death-stricken survivor mother: the lived experience of near miss mothers

**DOI:** 10.1186/s12978-021-01321-6

**Published:** 2022-01-10

**Authors:** Sedigheh Abdollahpour, Abbas Heydari, Hosein Ebrahimipour, Farhad Faridhoseini, Talat Khadivzadeh

**Affiliations:** 1 Student Research Committee, Mashhad University of Medical Sciences, Mashhad, Iran; 2 School of Nursing and Midwifery, Nursing and Midwifery Care Research Center, Mashhad University of Medical Sciences, Mashhad, Iran; 3 Social Determinants of Health Research Center, Mashhad University of Medical Sciences, Mashhad, Iran; 4 Psychiatry and Behavioral Sciences Research Center, Mashhad University of Medical Sciences, Mashhad, Iran; 5 Nursing and Midwifery Care Research Center, Mashhad University of Medical Sciences, Mashhad, Iran

**Keywords:** Maternal near miss, Lived experience, Qualitative study, Maternal morbidity

## Abstract

**Background:**

A Near Miss Mother (NMM) who survives life-threatening conditions, experiences intense physical, emotional, and psychological consequences following the maternal near-miss (MNM) events. The aim of this study was therefore to explore indepth understanding meaning of NMM everyday lived experiences on the social and cultural background of Iran.

**Methods:**

This qualitative study utilized a hermeneutic phenomenology procedure. The study was conducted in hospitals affiliated with the Educational, Research and Treatment Centerwhich usually handle the NMMs. The sampling was purposeful with a maximum variation of eleven NMMs. Datawere collected using unstructured face-to-face interviews, and thetranscribed data were analyzed using Diekelmann, Allen, and Tanner’s seven-stage thematic analysis approach.

**Results:**

"Death-stricken survivor mother" was the central emerged theme, and three extracted sub-themes included: "Distorted psyche on a journey to death", "physical destruction due to an ominous event ", and the "vicissitudinous life after reviving ". These sub-themes, in turn, involved 12 sub-themes that emerged from 38 common meanings and 1200 codes.

**Conclusions:**

Findings demonstrate that the living conditions of NMMs are mixed in all aspects of the MNM event. They need a supportive program that includes additional follow-up visits, psychological support from the time of hospitalization until long-time after discharge, alleviation of social, sexual, and financial worries to return them to the normal life, as well as psychosocial rehabilitation to increase their life quality. Furthermore, post-discharge care in NMMs should be done actively and directly at their homes.

## Plain language summary

A maternal near miss (MNM) is a life-threatening condition experienced by a mother with organ failure due to severe maternal morbidity. Near miss mother (NMM) experiences adverse outcome such as physical, emotional, and psychological consequences after near miss event. Understanding the meaning of these mothers' lived experience and listening to their voices will help in reducing the burden of complications and will be effective in rehabilitating the disability created in their life. "Death-stricken survivor mother" was the central emerged theme resulting from the study of phenomenology. The main body of this theme reflects the severe physical, psychological burden and vicissitudes life. Server maternal morbidity due pregnancy and childbirth, have made NMM mother's life very different from those of normal mothers. The living conditions of these mothers are mixed in all aspects of the MNM event. Long-term physical and psychological damages they have endured, should be on the agenda of women's health policy makers to support them for years after discharge with integrated care.

## Background

Near-Miss Mothers (NMM) are mothers who are in life-threatening conditions due to maternal morbidities and go close to death due to the failure of a vital organ, but survive [1]. The World Health Organization (WHO) states that the similarities, differences, and relationships between women who have died and those who have survived can be invaluable in identifying factors that contribute to improving the quality of care because both of them have common characteristics in a wrong process that leads to the maternal mortality [1, 2].

A global systematic review—in a large multicountry study revealed that based on WHO approach, the prevalence of MNM cases in health facilities was 18.67 per 1000 live births [3]. Per each maternal mortality case, an estimated of 20 mothers survive but they go on to experience the consequences of related morbidities, with long-term effects on their mental and physical health [4, 5]; however, no precise information is available about their lives after being saved from death. Most women who survive severe complications of pregnancy present a “maternal near-miss syndrome” This means that these mothers experience a cascade of physical and psychological symptoms such as fear and shock during the immediate emergency, anxiety, depression, post-traumatic stress[7–10]. ANMM case experiences intense physical, emotional and psychological consequences[9] and sensation of impending death[6, 10, 11]. However, there is a dearth of information about their long time experience of living under new circumstances following the MNM events.

Therefore, maternal health services should move beyond the focus on emergency obstetric care to a broader approach that encompasses preventive and early interventions, and integration to expand the narrow focus of maternal health, moving beyond surviving to thriving [12]. According to the conceptual framework for maternal morbidity, the condition negatively impacts on the woman’s wellbeing and/or functioning [13], and calls for “rethinking maternal health” using a life cycle or life-course [14], which should be a priority for women's' health research.

Qualitative study such as phenomenology, is most useful when the goal is to go beyond knowledge of core concepts and essences to interpret contextualized human experiences[15]. According to Willig, phenomenology as a method of qualitative research is a discipline that focuses ‘on people's lived experience of the world in which they live and what it means to them[16]. To understand the near miss phenomenon, it is necessary to achieve meaning by interpreting mothers' life experiences in the socio-cultural context of their lives [17].

Given that in Iran, for every thousand live births, there is one NMM [4], ignoring the life experience of such women, we will face an increasing trend of mothers who will suffer from burden of complications. Noticing the complexity ofthe burden of physical and psychological complicationsand regarding the lack of phenomenological studies for a deep understanding of the long-term experience of NMM h in the social and cultural background of Iran, the aim of this study was, therefore, to explore and gain an in-depth understanding of the meaning of everyday lived experiences of "maternal near miss" phenomenon.

## Methods

In order to gain insight into everyday NMMs’ lived experiences after discharge, a qualitative research design involving semi-structured interviews was chosen. Individual interviews were conducted in order to gain an understanding of NMMs’ experiences. The study was guided by Diekelman’s methodological interpretive approach, which is based on the phenomenological philosophy of Heidegger to provide an understanding of lived experiences [18]. Therefore, Heidegger’s phenomenology was applied to address the situatedness of individual’s dasein in relation to the broader social, political, and cultural contexts[15, 19]. Based on this method, we have investigated NMM experience of health in the context of family traditions, community values, and the broader sociopolitical context.

### Participants and study setting

The qualitative study took place in Iran. The sampling method in this study was purposeful with the maximum variation. For this purpose, mothers were selected from the hospitals affiliated with Educational, Research and Treatment Center, which usually handle the NMMs. These hospitals are the referral hospitals in the province that have over 3,000 deliveries per month and in terms of facilities and equipment, accept mothers who suffer from severe maternal morbidities. The mothers' information was extracted from their electronic records, and then appointments with them were arranged. The interview setting was selected according to the convenience of the participants, such as their own homes. Interviews were conducted at least one year after discharge from hospital, so that mothers could have experienced new conditions in everyday life after the MNM event. The inclusion criteria were NMMs who experienced an organ failure according to WHO criteria. Exclusion criteria included being unable to speak or hear and unwillingness to continue cooperation in the research. After a mother entered the study, a written consent was obtained before the interview.

Sampling took place from January to December 2019. By reviewing the documents of the maternity ward, the names and details of eligible mothers were extracted from their electronic files. Approximately one year after discharge, the fifteen selected mothers were contacted through telephone call by the first author. Thirteen mother gave their consent to participate in the interview. Two of the mothers were excluded from the study because they did not have the ability of speaking and hearing following recent MNM events. Finally, eleven interviews were conducted, the summery of theire characteristics are shown in Table 1, but due to the principle of confidentiality of the participants, it is not possible to mention all the details. Ethical issues were considered and necessary information was given about the aim of the study, and the voluntary participation in the study. Moreover, the length of the interview was determined by participants.

**Table 1:** Demographic information of study participants

Participant	Type of organ failure	Mother's job	Husband's job	Number of previous children	Education
1	Uterine Failure—Hematology	Housewife	Insurance agent	2	Diploma
2	Uterine dysfunction	Housewife	Electrical engineer	1	Bachelor
3	Neurological failure	Housewife	Self-employed	1	Diploma
4	Neurological failure	Housewife	Health worker	0	Diploma
5	Uterine and bladder failure s	Housewife	Hospital paramedic	0	Middle School degree
6	Respiratory failure / preeclampsia / hematological failure / advanced lung cancer	Housewife	Locomotive driver	1	Bachelor
7	Uterine dysfunction / hematological failure / gastrointestinal failure	Employed	Baker	1	Middle School degree
8	Cardiovascular Failure / Respiratory Failure / Renal Failure	Housewife	Self-employed	0	Bachelor
9	Kidney failure / hematological failure	Housewife	Driver	2	Diploma
10	Kidney failure / hematological failure	Employed	Worker	1	Bachelor
11	Kidney failure / hematological failure	Housewife	Painter	0	Middle School degree

### Data collection

One 70-to-100-min semi-structured in-dept interview with each participant, was conducted by the first author. In line with the phenomenological approach, both open-ended and flexible questions were propped to describe their everyday lived experience. Prompts such as “Could you tell me more about that” or “Could you give an example of this” were used to encourage the mothers to tell more. During the interview, their behavioral and nonverbal moods were recorded using field notes and kept in a personal diary. During the interview and data collection, the researcher paid attention to "bracketing" her ideas and mental assumptions [20].

The interview guide included the following: Please explain how did you experience near miss event? Please describe your experience after the discharge from hospital? Please describe to me how did you manage your everyday life at home. How do you experience in the new condition after discharge? After a presentation of the interview guide by the interviewer, the participants were invited to talk freely and in detail about their experiences. Sampling continued until data saturation [21].

In order to bracket the researcher’s preconceptions, and prevent the influence of the study findings, the first author wrote down her understanding of NMM [22]. Interviews were conducted in Persian and translated into English after analysis. All of the interviews were audiotaped and transcribed verbatim by the first author and a project assistant.

### Data analysis

Based on the Heideggerian beliefs, and Diekelmann, Allen, and Tanner’s guidelines [18] a step-by-step process was followed to analyze the narrative texts by the interpretive team. The analysis was typically done in seven steps: (a) reading the interviews to obtain an overall understanding; (b) writing interpretive summaries (c) analyzing selected transcripts as a group to identify themes; (d) member check to clarify disagreements in interpretation (e) comparing and contrasting texts to identify common meanings; (f) identifying patterns that link the themes; and (g) eliciting responses and suggestions on a final draft from the interpretive team. While performing each of the steps carefully, moving between the seven steps was done iteratively, from the overall impression to particular parts of the transcript, identifying themes and subthemes of NMMs’ perspective.

## Results

Among the eleven mothers participating in this study, 2 (18%) were employees and 9 (81%) were housewives. 4 (36%) were primigravida and 7 (63%) were multigravida. In terms of level of education, 4 (36%) had high school diploma, 3 (27%) had education less than high school diploma and 4 (36%) had bachelor’s degrees.

The Emergent central theme was " Death-stricken survivor mother " consisting of three subthemes: "Distorted psyche on a journey to death", "physical destruction due to an ominous event ", and the "vicissitudinous life after reviving ". These sub-themes, in turn, involved 12 additional sub-themes that emerged from 38 common meanings. The common meanings were obtained from 1200 codes (Table 2).

**Table 2:** Emergent Themes of Participant’s experience

The common meaning		The subtheme	The main theme	The initial theme
Death moment description		flashing moments of presence	Distorted psyche on a journey to death	Death-stricken survivor mother
React to unpredictable news		Encountering the Risk		
Encountering unpleasantreal events				
Perceived threat of danger				
Denying the existing reality		Outcome of the dispute with the death courier		
Forced progress on an irreversible path				
Stress				
Fear				
Feeling of guilt		Depression		
Despair				
uninterrupted crying				
Extended sadness and grief				
Feeling of loneliness				
Irritability and aggression				
Annoying thoughts about death		Post-traumatic stress		
Hyper-arousal of death symbols				
Avoid the shadow of death				
Physical destruction in moving towards death		Physical destruction due to an ominous event		
Persistent physical decadence				
Destruction of apparent acceptability				
Follow hard and exhausting treatments		Deviating from the direct road of everyday life	Vicissitudinous life after reviving	
Trying to solve psychological effects				
Late normal-life obtaining				
Disruption in performing every day activities				
Being dependent on the aid of others				
Trying to suppress the tragic reality of life				
Marital relations affected by the shadow of death	Damaged sexual intercourse	The shadow of disaster on those around the mother		
	Negative feelings regarding future childbearing			
	Emotional closeness			
	Negative psychological response of the spouse to the mother's illness			
Shading of the ghost of death on families				
A motherless child				
Anxiety about the uncertain future		Uncertainty of the future		
Concerns about the living conditions of loved ones after the death of the mother				
Disruption in making social connections		Social distancing		
Imposing exorbitant costs because of the disease		Sacrificing wealth for health		
Resorting to the infinite power		Surrendering to the divine fate		
Paying attention to spirituality to reach compatibility				

The subtheme "Distorted psyche on a journey to death" refers to experiences that have affected the psyche of mothers. Their psychological symptoms have been experienced as both of early and long-term symptoms. The subtheme "physical destruction due to an ominous event" refers to the multiple and complex physical experience whose negative consequences are frequently appearance in various organs of the body. The theme of " vicissitudinous life after reviving " refers to changes in the living conditions of mothers that have affected different aspects of their lives in every way.

### Distorted psyche on a journey to death

All NMM had experienced psychological complications. These morbidities happened at different times and locations when dealing with the situation. This theme consists of five further sub-themes. From a temporal perspective, these psychological reactions happen with transposition. Therefore, the subthemes of "Flashing moments of presence", "encountering the danger", and "Outcome of the dispute with the death courier ", are early psychological reactions that usually occur against near-death critical and hard situations. All mothers experienced a series of psychological reactions with long-lasting psychological impacts still persistent for up to a year after the event. Further, having "depression" and "post-traumatic stress disorder" was unsolved for the mothers.

The "flashing moments of presence" refer to the moments when the mother feels she is very close to death, and her presence and life are passing through at flashing speed: she finds herself in her final living moments. Every mother's experience from this moment was unimaginable and hard, so all mothers expressed that it is very difficult to describe it and coping with this position is very cumbersome. In this regard, a 19-year-old mother who suffered from cerebral hemorrhage and coma during pregnancy remarks:

"I put my clothes up, sat down on the floor, to cool down my body. Then, I was dizzy. It was like I was there, but my head was dizzy as if my brain was moving away from me. At that moment, I thought that I wouldn't live anymore and that was the last moment of life ". (P3)

The "encountering the danger" shows they experienced physchological impacts which were due to the conditions which exposed them to risks such exposures were often made up of "exposures with unpredictable news", "encountering unpleasant real events" and "perceived threat of danger".

Some mothers said that they had psychological reactions, such as shock and wishing to go unconscious in the face of unpredictable news concerning their problem. Furthermore, factors such as doctors' surprise at the mothers’ survival indicated to them that they were in a serious danger. A 24-year-old mother with abdominal distension and bowel perforation after emergency cesarean, says:

"Keep the mother here for a minute (with emphasis), you (the husband) are responsible for her death. She said every minute counts for her. She said 'I wonder how she has survived this long'". (P7)

The theme of "outcome of the fight with the death courier" suggests that thinking about death and the presence of the angel of death disrupted most mothers' thoughts after they go close to death and overcome e critical conditions and emergencies. This theme consists of the succeeding sub-themes of "rejecting the existing reality", "compulsory progression in the irreversible path", "stress" and "fear".

"Rejecting the existing reality" was described by a 31-year-old mother suffering from severe preeclampsia, kidney failure, and had to undergo dialysis forever as follows:

"I was saying to myself, 'All these people give birth, and there's no problem. Why should I have a problem? What's the reason? I couldn't take it'. (P8)

A mother with experience of fear and cardiopulmonary failure, who had to go to a well-equipped hospital in the city, says*:*

"It was a long way. I was scared, even though we came by plane. But they said it was your responsibility then. I was panicked at that moment. It was a pretty grave situation at that moment, and that horror is still in my mind."(P6)

The next subtheme that emerged from the long-term psychological impacts of most NMM' statements is "depression". Typical symptoms of depression, such as guilt, despair, uninterrupted crying, extended sadness and grief, loneliness, irritability, and aggression, are most evident in these mothers' life experiences. Here are some instances of mothers speaking about different symptoms of depression:

"I was crying and saying, 'I want to die, just let me die. Why are you doing this to me?'(P7)

"I was really shattered. It was incredibly hard for me. No matter who checked on me or what happened; I would only cry (she cried). I couldn't control myself at all and felt very bad."(P1).

"Well, it's really hard to be alone (she cries). Well, I felt lonely in the hospital for some days. It was tough for me, and I felt abandoned."(P6)

"I feel so guilty for not choosing a good hospital for childbirth, and I blame myself."(P11)

Another long-term psychological complications, derived from many participating mothers' experiences, was "post-traumatic stress". This theme emerged in three sub-themes, namely "disturbing thoughts about death", "hyper-arousal of death symbols" and "avoiding the shadow of death". A 43-year-old mother who had a hysterectomy due to maternal morbidity presented her experience of disturbing thoughts*:*

"After removing the uterus, I thought it was going to happen again with the slightest bleeding. When I went to the doctor, he would say, 'you no longer have a uterus. You have had it removed. What are you afraid of now?' And I would tell him, 'I can't help with it, I imagine the same thing is going to happen again'. (P1)

Another mother with a two-month history of intensive care in the hospital due to kidney failure with hyper-arousal of death symbols experience says*:*

"For example, just this week, my mother-in-law is going to have her back operated. The thought of those angiocathes, ampoules, doctors, nurses, and their behavior ravages me. I cried so much, I was even more worried for her than even the others and her children". (P3)

Avoiding the shadow of death was displayed by many mothers as avoiding what is reminder of death. In this respect, a mother with a history of three times abdominal laparotomy and then a hysterectomy says:

"I avoid seeing myself in the mirror. I don't want to look at my stomach now because it looks so bad. For example, I hate myself every time I go to the bathroom. I try not to see my belly in the mirror". (P7)

### Physical destruction due to the ominous event

In this research, mothers who almost died but survived suffered from short-term and long-term maternal morbidities, which were constantly recurred in their lived experience. Because these mothers have undergone life-saving interventions, including blood and platelet transfusions, repeated surgeries, prolonged hospitalization, hemodialysis, and other paraclinical procedures. Although survived, these mothers have experienced physical destruction and short-term and long-term injuries. Physical destruction due to the ominous event theme implies that the tragic death accident is intermingled with the positive birth event. This theme emerges in three sub-themes, including "Physical degeneration in the path of death", "Persistent Physical decadence," and "Losing Apparent Acceptance".

"Physical degeneration in the path of death" depicts the first physical failures that happened short time from MNM event. A mother who has been bedridden in ICU with cardiac and kidney failure for a long time expressed her experience of physical degradation:

"I would sleep in sitting positions and couldn't breathe at all. They had to keep giving me an oxygen mask all the time. Until one day, they consulted with the cardiology section, and they took me there for an echocardiography. There, they came to realize that only 17% of my heart worked, and it could stop beating at any moment. They quickly said that I must be moved to the cardiology section. Then I was in the cardiology ICU. I was there for three weeks". (P8)

"Persistent physical decadence" indicates that persistent physical morbidity such as hearing or memory loss or organ failure remain a mother's lifetime physical burden. A mother who has been in ICU for one month because of neurological failure and coma says:

"I can't walk now. I have no balance. I have no eyesight either. I am blind now. I couldn't see anywhere after recovery. I had lost my short-term memory too and now I'm starting to remember little by little"..(P4)

"Losing Apparent Acceptance" means that in addition to organ failure, the mother does not have the previous acceptance in terms of appearance and aesthetics, and persistent physical morbidities have affected her physical beauty. A mother who is currently experiencing abdominal dialysis owing to kidney failure says:

"I have now sewed a cloth and stretched its top and bottom for my abdominal catheter and skin to be unseen. I wear it permanently. I don't want anything to be seen. I wear a dress that hides my belly when I go to parties. After all, my body has changed in terms of aesthetics. It is very hard to see my appearance messed up like this".(P8)

### The vicissitudinous life after reviving

The experiences of the participants in this study show that not all mothers who came to the hospital intending to give birth return with the same previous conditions. Rather, they are discharged after experiencing the most severe and indescribable conditions, with new living conditions different from former ones. These issues force them to live a vicissitudinous life. This sub-theme itself has emerged in six further sub-themes, revealing the new conditions directing the mother's life. These sub-themes include "Deviating from the direct road of everyday life", "Shadow of disaster on mother's acquaintances", "Ambiguity of the future", "Social distancing", "Sacrificing wealth for health" and "surrendering oneself to divine fate".

The "Deviating from the direct road of everyday life" subtheme that was engaged in the all mothers lived experiences, was derived from the six common meanings mentioned in the following. One of these common meanings is the demand for permanent and exhausting follow-up of the therapeutic process that is tiring to the mother. A 23-year-old mother with neurological failure says:

"Well, it is hard for one to always be sick and keep going to the doctor and the hospitals. Until now, I've had three brain angiographies. These things put me off work and life". (P4)

Some other mothers stated it was quite time-consuming to return to normal life. Regarding the theme of "Late normal-life obtaining", a 43-year-old dialysis mother says:

"I would wash clothes, but I didn't feel like collecting them back. Dishes would be stacked, and house works remained undone. Until I got dialysis, things started to improve a bit. I got better and my energy recovered". (P10)

Most mothers stated that they could not perform normal life activities and that their physical and psychological changes had upset their everyday routine. A mother with a two-month history of coma says:

"I haven't cooked since then. But it's been about two weeks since I managed to take bath by myself. Also, it has been around a month that I can go to the bathroom by myself". (P4)

The "Shadow of disaster on mother's acquaintances" subtheme, derived from the statements of many mothers, designates that the bothersome incident that happens to the mother affects her husband, family, parents, and her previous children, its negative effects are imposed on those around her, unwantedly. The outcomes of this event directly affect her marital life. Some aspects of hurt marital relationships include sexual relationships, childbearing behavior and decisions, and psychological responses, such as depression and stress in the spouse.

A mother with a daughter and emergency hysterectomy, speaks of changing future childbearing decisions:

"My husband and I weren't on speaking terms for a while. Because he wants a boy and I have no uterus to have a baby. All our earlier arrangements to have a baby changed". (P11)

Spouse's negative psychological responses were manifested in different forms. A mother with an emergency hysterectomy experience says*:*

"My husband is depressed now. Insofar as when the name of the hospital comes up, he feels pretty bad. He doesn't want me to talk about those bad memories anymore". (P7)

The next sub-theme represents the concern of most mothers on "ambiguity of the future", one dimension of which is their fear of their unpredictable future. Most mothers were doubtful of a concerning future. A mother with former neurological failure and cerebral hemorrhage, says*:*

"I have another brain angiography. God willing, if I can get really, really well again, it's pretty good. But I don't know if I will or not" (P6)

The next theme, "Social distancing", depicts the social burden that this event puts on their lives, and they are unwilling to have healthy communication with other because of different physical and psychological reasons. In this regard, a 23-year-old mother says:

"My connections with others have become much less and I speak less. I am no longer in touch with my friends. I told them not to call me anymore because I don't feel like talking". (P3)

The other theme is "Sacrificing wealth for health": these mothers have spent all their money and financial savings of their marital life to achieve their previous health, and they have suffered an economic fatal blow. The incident imposes tremendous costs of hospital and paraclinical measures, tests, medication, and so on. A mother with a three times history of ICU says:

"We had a piece of land and a house and two cows. My husband sold them because of my situation. There was no more money left until my husband was forced to sell my gold without me knowing. Now my father is supporting us mostly". (P7)

The subtheme of "surrendering oneself to divine fate" indicates in mothers' experiences, surrender to fate has never been ignored and resorted to God to reach their previous health and wellbeing. A 23-year-old mother who had undergone hysterectomy following severe PPH speaks about her husband's reaction to her illness, *"When I was discharged from ICU, he was in Imam Reza shrine, sitting there until the very morning and crying and praying, 'Imam Reza, I want my wife from you'. (P7)*

Finally, the conclusion from the mothers' experience shows that all aspects of the lives of mothers who have suffered an MNM event due to maternal morbidity have been Death-stricken. Plus, encountering death and its adverse outcomes leave a negative psychological, physical, economic, and social experience for the mother, had been deviated her from the direct path of normal life. Outcomes of this research was new knowledge about the meaning of maternal near-miss phenomenon. This issue is important because in order to promote women's reproductive health, it is necessary to understand their maternal morbidity experiences and the necessary planning to reduce the burden of complications and their rehabilitation.

## Discussion

The present study is an interpretive phenomenological study aimed at undestanding the lived experience of NMMs a year after the MNM event. According to WHO criteria, these mothers had the experience of an organ failure. The central theme emerged was "Death-stricken survivor mother", which appeared in three main themes of " Distorted psyche on a journey to death", "physical destruction due to the ominous event " and "the vicissitudinous life after reviving".

One of the themes of this research, named "Distorted psyche on a journey to death", represents the psychological aspects of these mothers' difficulties. These mothers exhibited symptoms of psychological stress from encountering the moment of death, depression, and post-traumatic stress. This result is in line with the study by Abdollahpour, who examined the maternal lived experience of traumatic childbirth [9] because all NMMs have experienced a life-threatening condition and perceived their childbirth as traumatic. Also, in Elmir’s study in Australia in 2012, NMMs who had a life-and-death experience after an emergency hysterectomy reported symptoms of flashback and post-traumatic stress disorder after four years [23]. Souza in a study conducted in 2009 on the experience of Brazilian mothers hospitalized in ICU following maternal morbidity found that they had feelings of fear and hopelessness immediately after discharge [7]. Given the short time from MNM event, this matter is in line with the early results of the mothers' experiences in this study. It was predicted that if the two previous studies had investigated long-term morbidities, the symptoms of depression and post-traumatic stress disorder then would have been as clear as it is in the current study. Kay's study in 2014 in Uganda unveils a range of 'loss' emerging in mental, physical, economic and social contexts. Perhaps the reason why most of those results are similar to that of this study is the adoption of the phenomenological study method. By this method, the in-depth experience of mothers emerges in more depths [24].

The second theme that emerged in the present study was "physical destruction due to ominous event" in which the physical dimension of mothers was expressed as short-and long-term symptoms. Mothers have repeatedly stated the experiences they had suffered in terms of physical injuries. This theme was also mentioned in a study by Souza in 2009 as "physical morbidity of health-threatening conditions" [7].This result is also in line with the study of Cram in New Zealand in 2019, in which he examined the maternal experience of critical maternal morbidity after 6–12 months of delivery. It has also highlighted the physical dimension of receiving emergency and intensive and critical care [25].

The third theme of this study emerged as "the vicissitudinous life after reviving ". One perspective of this theme was the influence of the disaster on those around the mother, including her husband. In this study, feelings of mood changes, depression, and psychological disorder by the husband also proved to affect other aspects of marital life. In 2014 Hinton studied the experience of MNMs’ husbands in the United Kingdom, within the time-span close to the incident and several years after it. The husband felt powerless and shocked at seeing his wife's condition [26]. Consistent with these findings, the results of a study by Mballinda in 2015 on husbands' perceptions of MNM in Uganda 4–12 months after NMM event, indicated that in maternal critical situations, men experience a feeling of powerlessness, alienation, and loss that results in marital disruption [27]. Another study by Kay in the period of 6 months after the incident in Uganda on mothers who experienced uterine rupture showed that these mothers experienced impaired marital relationships and developed impaired reproductive behavior [24]. In the present study, in the theme of catastrophic shadow on the spouse, the mother clearly felt the psychological and temperament changes of the spouse Another theme of the study was "Sacrificing wealth for health", which indicated a fatal economic blow that, in line with Kay's study, led to unpleasant economic outcomes and a loss of revenue [24]. Another dimension of this theme in this study was social distancing and disruption in making social contacts, in line with Hinton's study in the United Kingdom on mothers who experienced life-threatening ailments [10].

Overall, given that the qualitative studies that have concentrated on the experience of MNMs are inadequate, not all aspects of the present study are comparable to those of other existing investigations. It is suggested that further examinations be carried on topics including short-term and long-term psychological, physical, social, economic dimensions, future childbearing, the influence of the incident on the mother's surroundings, heightened spirituality to reach adaptation, and so on.

The strengths of this study are as follows: First, this is the first study on NMMs in which the participants were selected based on WHO criteria. Second, this is the first interpretive phenomenological study which used the Dickman method and has deeply addressed the understanding of the meaning of the lived experience of NMM. Third, this is the first study which examined the long-run experience of mothers with a wide variety of organ failure types. One of the limitations of this study is the difficulty of communicating and setting appointments for interviews with mothers who were discharged from the hospital a year ago, especially mothers who had to come a long distance to attend the interviews. This was partially controlled by setting the appointment at mother's convenience.

## Conclusion

Severe complications of pregnancy and childbirth have made NMM mother's life very different from those of normal mothers. The living conditions of these mothers are mixed in all aspects of the MNM event. It is recommended that support programs be on the agenda of the health system and women's health policy makers as primary care.

It is recommended to design and implement support programs for NMMs, including additional follow-up visits, psychological support for mothers and other family members from the time of hospitalization until long-time after discharge, counseling about marital relations and sexual counseling, counseling with family members on helping the mother and reducing the assignment of the housework to her, alleviation of the mother's worries from returning her to the normal life, as well as providing her with psychosocial rehabilitation to increase her life quality. It is also recommended that integrated care of the hospital be continued a long time after discharge.

Furthermore, because of the long-term physical and psychological damages they have endured, it is better to take care actively and directly by visiting them at their homes.

Information about quality of care and the use of critical interventions gained by understanding the meaning of their life experience are useful for shaping improvements in health care and strengthening the contribution of health systems. So, health providers can develop strategies for preventions dealing with the problem and demonstrate ways of improving maternal wellbeing by studying related research.

## Data Availability

Data could be available upon a reasonable request and with the permission of Mashhad University of Medical Science ethical committee. The interviews used in this study are taken from a part of the doctoral dissertation work.

## Abbreviations

NMM: Near Miss Mother
MNM: Maternal near-miss
WHO: World Health Organization
